# Lifestyle Factors and Health Awareness for Improving Bone Density Acquisition in Adolescent Girls: A Pilot Study With Secondary Data

**DOI:** 10.7759/cureus.52150

**Published:** 2024-01-12

**Authors:** Kazue Yoshihara, Hiromi Kawasaki, Zhengai Cui, Sae Nakaoka

**Affiliations:** 1 Department of Health Science, Graduate School of Biomedical and Health Sciences, Hiroshima University, Hiroshima, JPN; 2 School of Humanities and Management, Guangdong Medical University, Dongguan, CHN

**Keywords:** exercise, feeding behavior, lifestyle, adolescent, stiffness, bone density

## Abstract

Objectives: Adolescent girls are in an important life stage of peak bone mass (PBM) acquisition. We aimed to examine the provision of health guidance for female high school students and elucidate the factors that can increase bone density.

Design: A quantitative, cross-sectional analysis was used in conducting the study.

Sample: Sixty Japanese female first-year high school students comprised the study sample.

Measurements: We included secondary data from a bone mineral density survey conducted among female high school students in August 2016 and analyzed in 2023.

Results: Thirty participants each (age, mean±SD, 15.80±0.45 years) were assigned to the high and low bone stiffness groups. The high bone stiffness group had higher health awareness, better lifestyle habits, and higher club activity-related stress than the low bone stiffness group.

Conclusions: Lifestyle factors and health awareness improved bone density in adolescent girls. Items related to exercise were particularly important for improving bone density. Eating habits and health awareness also affected bone density. Adolescent women should recognize that adolescence is a critical period for lifelong health, a period with few opportunities to develop health awareness. Acquiring appropriate lifestyle habits during adolescence can help prevent bone density loss.

## Introduction

Childhood and adolescent bone mass likely affects lifelong bone health [1,2]. Peak bone mass (PBM) is the amount of bone acquired before a stable skeletal state is achieved in young adolescents [3]. Furthermore, PBM is thought to be achieved during the first 20 or 30 years of life; however, the exact timing depends on the body site and sex [4]. The maximal bone mass increase in women occurs rapidly in the two to three years after menarche, with the total hip reaching PBM by the ages of 16-19 years [5].

Bone mass declines rapidly after menopause owing to decreased estrogen production. Increasing the PBM is crucial to prevent rapid bone loss and osteoporosis [4,6]. Additionally, PBM is influenced by genetic and environmental factors. The main environmental factors that affect bone mass density include physical activity, lifestyle, and dietary intake [7-10]. Lifestyle choices contribute to 20-40% of PBM in adults [3], and it is reportedly altered by nutrition and exercise [11]. Thus, an increase in PBM requires a lifestyle that positively impacts bone density, particularly during the first two to three years after menarche.

Two to three years after menarche, adolescent girls are junior high and high school students. Therefore, lifestyle habits related to bone density in formal education should be taught to improve PBM. Post menarche, junior high and high school students are generally healthy, and bone density does not significantly affect the health of these students. The significance of health guidance is frequently underestimated, and there is limited awareness of the importance of bone density-related health guidance. Schools attempt to solve other emerging issues by prioritizing them, namely, underage smoking and drinking, drug abuse, and sexually transmitted diseases. Owing to insufficient practical examples, the content and basis of health guidance for bone density in the first two to three years post menarche have not been published. Unfortunately, bone density screening is not recommended for adolescents; therefore, raising awareness of its potential problems is especially important [12].

In Japan, instructions on increasing bone mass are related to the prevention of postmenopausal osteoporosis. Osteoporosis significantly reduces the quality of life of older adults because it causes fractures that require care in daily living. Middle-aged people and older adults receive guidance on lifestyle habits to improve bone density. Moreover, lifestyle habits are closely related to cholesterol management. Dyslipidemia or elevated total and low-density lipoprotein cholesterol levels are associated with low bone mass and increased fracture risk [13]. Osteoporosis and dyslipidemia are lifestyle-related diseases that are caused by the cumulative effects of daily lifestyle habits, which should be addressed at an early stage. The management of lifestyle habits from childhood leads to the prevention of lifestyle-related diseases. Therefore, to prevent the occurrence of osteoporosis, which is a lifestyle-related disease, a lifestyle that increases bone density from a young age should be established. Among junior and senior high school students, health guidance related to bone density can enhance their understanding and recognition of the importance of lifelong lifestyle and health management as well as enable the acquisition of high PBM. Understanding the underlying mechanisms acting on bone mineral density can help prevent bone loss throughout life. Increased bone mineral density in junior and senior high school students prevents osteoporosis in middle-aged and older adults. Thus, instructing post-menarchal junior and senior high school students about bone density constitutes a highly significant population-based approach to preventing osteoporosis.

Lifestyle habits, including proper physical activity, lifestyle, and diet, are influenced by health awareness. Health-related behavior is based on individual awareness of health, and adolescents who have a more positive perception of adopting healthy lifestyle behaviors are more likely to demonstrate these behaviors [14]. Lifestyle habits vary according to differences in health awareness, such as being cautious about one's health, which may constitute one of the factors that affect bone density. Psychological stress leads to complex physiological and behavioral changes affecting bone health [15]. To improve bone density, besides educating junior and senior high school students on lifestyle habits related to bone density, it is important to clarify the relevance of health-related awareness and psychological conditions (school stress) that are unique to bone mineral density during adolescence.

Many studies have investigated bone density and lifestyle habits; however, only a few have investigated the factors, including lifestyle habits, health awareness, and psychological characteristics, affecting bone density in high school students. Therefore, in this study, we targeted adolescent girls in an important life stage of PBM acquisition and examined the provision of health guidance for female high school students. This was done by clarifying the factors that contribute to increased bone density through desirable lifestyle habits, health awareness, and psychological conditions.

Bone density testing is not actively promoted among adolescent girls because they are generally healthy. Adolescent girls are minors and require parental consent. Therefore, it is not easy to conduct bone density studies and interventions in adolescent girls. In this study, a pilot study was conducted using secondary data from health examinations at High School A to provide material for future research.

## Materials and methods

Design

This cross-sectional study involved a secondary analysis of data obtained from a bone mineral density survey conducted as part of a health checkup in August 2016 at High School A, a private girls' high school located in Hiroshima, Japan, that undertakes many extracurricular activities [16]. The health screening measurements and surveys were conducted at the same time. Several students with a sports endorsement live in dormitories and are members of athletic clubs.

Sample

The participants were 60 first-year high school students of High School A who participated in the questionnaire survey and underwent bone density measurement. The analysis was conducted in 2023. The Strengthening the Reporting of Observational Studies in Epidemiology cross-sectional guideline was used as a reporting checklist for the cross-sectional studies [17].

Ethical considerations

This study was conducted in accordance with the tenets of the Declaration of Helsinki and was approved as an epidemiological study by the Hiroshima University Ethics Review Board (approval number: E-562-2). With the permission of the principal, data from the survey that was conducted as part of the health checkup of High School A were included in a secondary analysis. Responses were processed and used after randomized de-identification to protect participant confidentiality.

Measures

We surveyed the following aspects: (1) basic attributes (age, living conditions, presence of diseases, history of fractures, club activities, and lifestyle habits), (2) exercise status, (3) dietary intake status, (4) knowledge of bone density, (5) lifestyle-related behaviors and health awareness, (6) school stress, and (7) menstrual status. The measured items included bone density (bone stiffness), height, weight, and subcutaneous fat thickness.

For ascertaining exercise status, we used the physical activity index developed by Hashimoto [18] to quantify the amount of physical activity. Exercise intensity was rated on a 5-point scale (from 0 points: no exercise to 4 points: extremely strenuous exercise). Duration of exercise (daily) was rated on a 6-point scale (from 0 points: no exercise to 5 points: ≥90 minutes of exercise). The frequency of exercise was rated on a 6-point scale (from 0 points: no exercise to 5 points: almost every day). These scores were multiplied to obtain the amount of physical activity. To determine the motor behavior change stage [19], we used the motor behavior change stage scale developed by Oka, which comprises items that measure the actual motor behavior from the past to the present and the state of motivational readiness for the motor behavior. The contents of each item are as follows: indifference (1 point), interest (2 points), preparation (3 points), execution (4 points), and maintenance (5 points) periods. The 5-point scale identifies the motor behavior change stage of each individual.

Regarding dietary intake, we used the comprehensive evaluation of nutritional intake based on a food intake frequency questionnaire developed by Tsukahara et al. [20]. A questionnaire survey was conducted to elucidate dietary habits, and the frequency of intake for each item (eaten every day: 4 points; rarely eaten: 1 point) was scored and evaluated.

Eight questions about knowledge of bone density were included; correct answers were counted as 1 point, and incorrect answers were assigned 0 points.

Behaviors related to lifestyle habits were considered related to bone density [3,9,21,22]. Nine items (never: 1 point; often: 4 points) were created for health habits, and three items (disagree: 1 point; agree: 4 points) were created for health awareness. Although it was not an extant scale because the data of the school health checkup were analyzed for this secondary purpose, statements such as "I am conscious about my health," "I want to actively obtain information about my health," and "I consult an adult such as a parent or teacher when I have concerns about my physical condition" were considered as responses that indicated health awareness.

School stress was rated on a 4-point scale for the frequency of school stress experience against 25 school stressor scale items [23]. The school stressor scale comprises the following five factors: "relationship with teachers" (five items), "schoolwork" (five items), "relationship with friends" (five items), "club activities" (five items), and "school rules and regulations" (five items).

Menstrual conditions were investigated in terms of age at menarche, menstrual cycle, presence of amenorrhea, and duration of amenorrhea.

Bone density was measured using an ultrasonic measuring device (A-1000 InSight, GE HealthCare, Chicago, Illinois, United States) exclusively for the calcaneal bone; subsequently, the right calcaneal bone density (stiffness index) was measured. Although the bone stiffness value is not a direct measure of bone mass, it is an indicator of bone structure and elasticity. However, it is commonly used as an indicator of bone mineral density in large-scale medical checkups, and the calcaneal bone stiffness value and single-energy X-ray absorptiometry (SXA) have generally been used to measure bone mineral density [24]. A high correlation between calcaneal bone density values obtained using SXA and calcaneal bone stiffness has been reported [25]. The thickness of the subscapularis (SS) and triceps (TR) skinfolds were measured using a subcutaneous fat tissue depth measuring device; the measurements from the two locations were added to obtain the subcutaneous fat thickness.

Analytic strategy

Students who participated in both the questionnaire survey and the measurement of bone density (bone stiffness) were included in the analysis. To clarify the factors that affect bone density in high school girls, based on the median bone stiffness results, the students were first categorized into groups with high and low bone stiffness and then compared in terms of lifestyle and perception. Bone stiffness measurements showed a normal distribution with 30 participants each in the low and high bone stiffness groups. The median bone stiffness was 100.50. Two students with bone stiffness <77 received health guidance from school nurses. Regarding lifestyle habits, knowledge about bone density, stress, and other variables were comparatively evaluated in the high and low bone stiffness groups using the chi-squared, unpaired t-, Wilcoxon rank-sum, and Kruskal-Wallis tests. Statistical analyses were performed using IBM SPSS Statistics for Windows, Version 29.0 (Released 2022; IBM Corp., Armonk, New York, United States). Statistical significance was set at p<0.05.

## Results

The participants were first-year high school students (age, mean±SD, 15.80±0.45 years), with 30 participants each in the low and high bone stiffness groups. Table 1 shows the characteristics of the participants who were included in the analysis dataset.

**Table 1:** Student overview and comparison of the low bone stiffness and high bone stiffness groups Analysis: chi-squared test

Characteristics			Student (n=60)		Low group (n=30)		High group (n=30)		p-value
			n	%	n	%	n	%	
Living	With parents	Yes	36	61	20	69	16	53.3	0.288
		No	23	39	9	31	14	46.7	
	With father	Yes	0	0	0	0	0	0	
		No	59	100	29	100	30	100	
	With mother	Yes	7	11.9	3	10.3	4	13.3	1.000
		No	52	88.1	26	89.7	26	86.7	
	In the dormitory	Yes	11	18.6	2	6.9	9	30	0.042
		No	48	81.4	27	93.1	21	70	
	With parents and grandparents	Yes	1	1.7	1	3.3	0	0	0.492
		No	58	98.3	29	96.7	30	100	
	With other people	Yes	4	6.8	3	10.3	1	3.3	0.353
		No	55	93.2	26	89.7	29	96.7	
Disease under treatment		Yes	5	8.5	3	10.3	2	6.7	0.671
		No	54	91.5	26	89.7	28	93.3	
Fracture experience		Yes	19	31.7	9	30	10	33.3	1.000
		No	41	68.3	21	70	20	66.7	
Club activities		Sports club	30	50	7	23.3	23	76.7	<0.001
		Cultural club	18	30	14	46.7	4	13.3	
		No club	12	20	9	30	3	10	
Experience of amenorrhea		Yes	12	21.4	7	24.1	5	18.5	0.748
		No	44	78.6	22	75.9	22	81.5	

Most students lived with their parents, except for 11, who lived in the dormitory. Significantly more students in the high bone stiffness group than in the low bone stiffness group lived in dormitories. No significant intergroup difference was found for the presence of disease during treatment or history of bone fracture. In the high bone stiffness group, significantly more students belonged to athletic clubs. No significant difference in participants with amenorrhea was observed between the high and low bone stiffness groups.

Table 2 presents a comparison of the high and low bone stiffness groups.

**Table 2:** Comparison of the high and low bone stiffness groups in terms of lifestyle factors and health awareness M: mean; SD: standard deviation; &: unpaired t-test or Wilcoxon rank-sum test; #: Kruskal-Wallis test

		Low group (n=30)		High group (n=30)		p-value	
		M	(SD)	M	(SD)		
Measurement items	Weight	51.47	(6.81)	56.04	(9.8)	0.045	＆
	Height	156.12	(5.21)	158.47	(4.6)	0.069	＆
	Subcutaneous fat thickness	31.73	(7.47)	34.18	(10.84)	0.403	＆
Necessity of improving lifestyle habits	Need to improve eating habits	1.9	(0.76)	2.33	(0.8)	0.032	♯
	Need to improve exercise habits	1.6	(0.67)	2.42	(0.68)	<0.001	♯
	Need for improvement in sleep weeks	1.67	(0.71)	1.83	(0.75)	0.378	♯
Exercise	Motor behavior modification stage	3.53	(1.72)	4.37	(1.22)	0.036	♯
	Amount of exercise	21.7	(24.82)	51.34	(28.34)	<0.001	＆
Nutritional intake	Nutritional intake status	13.29	(2.31)	14.54	(3)	0.087	＆
	Intake of green leafy vegetables	3	(0.83)	3.4	(0.09)	0.029	♯
	Intake of other vegetables (cabbage, cucumber, and onion, among others)	3.3	(0.84)	3.33	(0.92)	0.713	♯
	Intake of mushrooms	2.23	(0.9)	2.53	(0.86)	0.181	♯
	Intake of seaweed (hijiki and cloud ear mushroom, among others)	2.03	(0.93)	2.3	(0.92)	0.316	♯
	Intake of sugary drinks	2.33	(1.03)	2.3	(0.92)	0.925	♯
	Intake of sweet snacks	2.5	(1.01)	1.77	(0.82)	0.005	♯
	Intake of instant food, fast food, and side dishes	2.07	(0.74)	1.97	(0.96)	0.503	♯
	Intake of nutritional tonics	1.3	(0.65)	1.23	(0.57)	0.717	♯
	Intake of sweet drinks (during club activities)	0.1	(0.45)	1.36	(1.63)	<0.001	＆
	Intake of sweet drinks (not during club activities)	1.29	(1.06)	0.86	(0.81)	0.145	＆
Knowledge of bone density	Score of questions on bone density knowledge	6.03	(0.91)	6.53	(0.94)	0.041	＆
Lifestyle-related behavior	Exercising for 20 minutes or longer at least twice a week (other than physical education)	3	(1.29)	3.63	(0.76)	0.067	♯
	Doing vertical weight-bearing exercises (exercises that defy gravity, such as jumping and bouncing, in addition to physical education)	2.03	(1.01)	3.2	(1.03)	<0.001	♯
	Eating a well-balanced diet	2.93	(0.83)	3.33	(0.8)	0.046	♯
	Be aware of additives in food and drink	2.07	(0.74)	2.43	(1.1)	0.261	♯
	Conscious intake of calcium and vitamin D	1.93	(0.74)	2.67	(0.92)	0.002	♯
	Following a diet	1.87	(1.04)	1.77	(0.86)	0.886	♯
	Irregular bedtime	2.87	(0.94)	2.9	(0.71)	0.905	♯
	Getting enough sleep	2.43	(0.97)	2.47	(0.94)	0.834	♯
	Checking menstrual cycle	1.93	(1.11)	2.1	(1.03)	0.413	♯
Health awareness	I am conscious of my health	2.33	(0.84)	3.03	(0.93)	0.004	♯
	I want to actively obtain information about my health	2.77	(1.07)	2.93	(0.98)	0.573	♯
	I consult an adult, such as a parent or teacher, when I have concerns about my physical condition	2.6	(1.07)	2.83	(1.09)	0.378	♯
Menstruation	Age at menarche	12.21	(1.11)	12.28	(1)	0.820	＆
	Menstrual cycle	1.42	(0.5)	1.43	(0.51)	0.901	♯
Stress	Relationship with teacher	10.3	(5.07)	10.24	(4.68)	0.939	♯
	Schoolwork	15.8	(3.98)	15.14	(4.51)	0.691	♯
	Friends	8.43	(3.65)	7.79	(3.2)	0.604	♯
	Club activities	7.83	(3.87)	11.41	(4.78)	0.005	♯
	School rules and regulations	11.62	(5.63)	10.45	(5.04)	0.291	♯

Among the measured variables, participants in the high bone stiffness group weighed significantly more than those in the low bone stiffness group. Regarding the items considered necessary for improving lifestyle habits, significantly fewer students in the high bone stiffness group felt a need to improve their eating and exercise habits. In the exercise behavior change stage, significantly more students from the high bone stiffness group were already exercising. The amount of exercise, and, therefore, physical activity, was significantly higher in the high bone stiffness group than in the low bone stiffness group.

Regarding the nutritional intake, no significant difference was found between the high and the low bone stiffness groups in a comprehensive evaluation of nutritional intake based on the data obtained from the food intake frequency questionnaire. The frequency of the intake of green and yellow vegetables was significantly higher, and that of sweet snacks was significantly lower, in the high bone stiffness group than in the low bone stiffness group. Despite showing no significant difference in the intake of sweet drinks outside of club activities and during club activities, the high bone stiffness group had a significantly higher intake of sweet drinks than the other group. No significant difference was found in intake of nutritional tonics between the high and low bone stiffness groups.

Regarding behaviors related to lifestyle habits, significantly more participants in the high bone stiffness group stated "I am consciously taking calcium and vitamin D," ate well-balanced meals, exercised with a vertical load system, and believed that they were paying attention to their health.

The high bone stiffness group experienced higher club activities-related stress than the low bone stiffness group. No significant difference was found in stress factors related to relationships with teachers, schoolwork, friends, and rules and regulations in the study groups. Knowledge scores were significantly higher in the high bone stiffness group.

## Discussion

To protect women's lifelong health, it is important to examine bone density-related factors, such as adolescent women's health awareness, psychological state, and lifestyle behaviors.

The results showed that the items related to exercise affected bone stiffness, among others. The study center, High School A, is a high school with many extracurricular activities. Significantly more students belonged to dormitories and athletic clubs in the high bone stiffness group than in the low bone stiffness group. Generally, physical activity is important for a healthy lifestyle. Bone density is directly dependent on the life stage and age at the initiation of physical activity: prepubescence, early puberty, adolescence, young adulthood, and mature adulthood [26]. As PBM is achieved at approximately young adulthood, physical activity during that period may constitute a major factor in increasing bone density. Club activities are the main source of sports activities for young people in Japan and are positioned as educational activities that are developed in elementary, junior high, and high schools. Club activities frequently start from junior high school, and it is presumed that students join the same competitions from this point onward. Exercise habits initiated and continued from pre-adolescence affect bone density. A similar association was demonstrated in this study. In Japan, club activities are encouraged and popular at school. Continuing exercise in extracurricular activities is important for improving bone density; however, it has been highlighted that Japanese people tend to "withdraw from sports" when they enter high school [27]. In the absence of membership to a sports club, high school students rarely engage in new physical and sports activities; hence, it is necessary to make efforts to encourage students to engage in sports activities at an early age. In Japan, efforts are underway to shift from school-based to community-based activities against the backdrop of declining birth rates and improved teacher workloads to ensure that children have the opportunity to be involved in sports even in the face of declining birth rates. In Japan, most junior high school students and older students participate in sports through organizations attached to their schools, and community sports are focused mainly on elementary school students. On a global scale, the following three types of sports venues for the youth exist: where community sports are the main venues, where schools are the main venues, and where both community sports and schools are involved [28]. In the United States, where both community sports and school sports venues are offered, community sports activities are held throughout the year for children, allowing them to experience various community-based sports activities after school and on holidays [29]. In the future, physical activity and participation in sports should be encouraged among adolescents not only in schools but also in the community in Japan.

The amount of exercise was higher in the high, rather than in the low, bone stiffness group. Students in the high bone stiffness group were significantly more active in the behavior of "performing a vertical weight-bearing exercise (other than physical education)." Weight-bearing exercises, including jumping, affect bone density [30]. The results of this study were similar to those of previous studies. When focusing on physical activity, attention to the content of the exercise and health guidance is essential.

The high bone stiffness group had higher club activity-related stress than the low bone stiffness group. High bone stiffness is not caused by high stress related to club activities; rather, high bone density is a result of hard work in club activities, which may generate stress related to club activities. As school-related stress in high school students did not negatively affect bone stiffness, stress in adolescent women did not affect bone stiffness in this study. Therefore, more research is needed to determine how psychological factors affect bone density. The high bone stiffness group was significantly heavier. A close relationship exists between the growing bone mass and body weight, and bones are affected by mechanical loads due to the body weight [31-33]. A study on middle-aged people and older adults showed that metabolic syndrome was negatively associated with osteoporosis [10]. Although no significant difference exists between the high and low bone stiffness groups in the behavior of "I am on a diet," weight loss and rapid weight loss, particularly from dieting, have been reported to be associated with decreased bone mineral density, which does not fully recover when the weight is regained [30]. A higher body weight is a positive factor for bone health; however, excess weight may not be beneficial. In a study of children, moderately obese (body mass index (BMI) for age ≥95th and <1.2×95th percentile or BMI ≥30 kg/m^2^ and <35 kg/m^2^) and extremely obese (BMI for age ≥1.2×95th percentile or BMI ≥35 kg/m^2^) children had increased odds ratios (ORs) of lower extremity fractures, including foot fractures [34]. Extremely high BMI increases the risk of bone fracture, which suggests a decrease in bone quality and the risk of falls [32,35,36]. Therefore, proper weight management is important for bone health.

Regarding lifestyle habits, significantly fewer students in the high bone stiffness group than those in the low bone stiffness group thought they needed to improve their eating and exercise habits. Significantly more students in the high bone stiffness group than those in the other group answered "I am cautious about my health." The high bone stiffness group may not perceive the necessity to improve their eating habits or exercise habits because they are usually cautious. Moreover, the high bone stiffness group had a higher knowledge score of bone density than the low bone stiffness group. Possibly, several students in the high bone stiffness group were taking care of their health and gaining health-related knowledge. Lifestyle habits in adolescence persist into later adulthood. For adolescent women, health guidance that focuses attention on their health is useful to help develop health awareness.

The students with high bone stiffness were significantly more likely to state "I am consciously taking calcium and vitamin D." To date, several studies have reported the relationship between calcium intake and bone density. A previous study of junior high and high school students demonstrated that calcium supplements showed a significant difference in bone density [37]. Consuming dairy products, such as milk, is a convenient means to ingest calcium. Studies targeting adolescents have reported that the intake of dairy products, such as milk, increases bone density [38]. In contrast, other studies showed no direct relationship between milk intake and bone mineral density or bone fracture risk [39,40]. Notably, osteoporosis prevention requires the balancing of various associated factors and not simply the intake of an adequate amount of calcium [41]. Some studies have reported that zero milk intake and high dairy product consumption are associated with an increased fracture risk [42]. In this study, participants in the high bone stiffness group were more conscious about calcium and vitamin D intake than those in the other group, which implies that the intake of calcium and vitamin D is one of the factors influencing bone density in adolescent women. Through detailed research, we believe that a specific instruction method for calcium intake is necessary. In addition to calcium and vitamin D, the study identified several dietary intake factors that improve bone density. The frequency of the intake of green and yellow vegetables was significantly higher in the high bone stiffness group. Dietary habits are closely related to bone density. Focusing on the dietary habits of adolescent women before they achieve their PBM is crucial. The intake of green leafy vegetables has a positive effect on bone health [43]. To improve bone density, it is critical not only to eat a well-balanced diet but also to add health guidance, such as the specific intake of green and yellow vegetables. Studies have shown that the high intake of carbonated beverages and snack foods affects bone health in adolescents [37]. In this study, the frequency of the intake of sweet snacks was significantly lower in the high bone stiffness group than in the other group, which supports the results of previous studies. The intake of snacks may affect bone density in adolescents. Snacks can be regulated freely for adolescent women, unlike the three main meals. Self-care of female high school students regarding bone density can be facilitated through health guidance on eating snacks and their nutritional content. The intake of sweet drinks during club activities was significantly higher in the high bone stiffness group. In this study, as well as in previous studies, participation in sports was associated with increased consumption of junk food (sugar-sweetened beverages) [44], suggesting that specific adverse effects associated with extracurricular activities exist. High school students should understand which appropriate snacks and beverages can improve bone density [45].

This study had some limitations. The study sample was limited owing to the use of a single high school health checkup for secondary purposes. Thus, it will be necessary to conduct a large-scale survey in the future. As this was a cross-sectional study, causality could not be clarified. Future research should clarify the causal relationship between bone density and related factors and consider potential confounders such as genetic predisposition and social factors in addition to psychological, physical, and lifestyle factors. In the future, it will be necessary to not only examine the important factors for improving bone density that were clarified in this study but also ascertain the detailed content of health guidance and implement and evaluate actual health guidance.

## Conclusions

This study identified factors, such as lifestyle and health awareness, which are critical to improving bone density in adolescent women. Items related to exercise were particularly important in improving bone density, which was also found to be associated with diet and health awareness during adolescence. Despite the fact that adolescence is a critical time for lifelong health, the general healthy adolescent female population does not easily recognize the significance of health guidance, and few opportunities exist to foster health awareness. Increasing opportunities for health awareness, as well as acquiring lifestyle habits that increase bone density, can be a foundation for lifelong health.

## References

[1] Rozenberg S, Body JJ, Bruyère O (2016). Effects of dairy products consumption on health: benefits and beliefs--a commentary from the Belgian Bone Club and the European Society for Clinical and Economic Aspects of Osteoporosis, Osteoarthritis and Musculoskeletal Diseases. Calcif Tissue Int.

[2] Jürimäe J, Gruodyte-Raciene R, Baxter-Jones AD (2018). Effects of gymnastics activities on bone accrual during growth: a systematic review. J Sports Sci Med.

[3] Weaver CM, Gordon CM, Janz KF (2016). The National Osteoporosis Foundation's position statement on peak bone mass development and lifestyle factors: a systematic review and implementation recommendations. Osteoporos Int.

[4] Rozenberg S, Bruyère O, Bergmann P (2020). How to manage osteoporosis before the age of 50. Maturitas.

[5] Berger C, Goltzman D, Langsetmo L (2010). Peak bone mass from longitudinal data: implications for the prevalence, pathophysiology, and diagnosis of osteoporosis. J Bone Miner Res.

[6] (2023). Guidelines for the Prevention and Treatment of Osteoporosis. http://www.josteo.com/ja/guideline/doc/15_1.pdf.

[7] Karlsson MK, Rosengren BE (2020). Exercise and peak bone mass. Curr Osteoporos Rep.

[8] Proia P, Amato A, Drid P, Korovljev D, Vasto S, Baldassano S (2021). The impact of diet and physical activity on bone health in children and adolescents. Front Endocrinol (Lausanne).

[9] Swanson CM, Kohrt WM, Buxton OM, Everson CA, Wright KP Jr, Orwoll ES, Shea SA (2018). The importance of the circadian system & sleep for bone health. Metabolism.

[10] Chen PH, Lin MS, Huang TJ, Chen MY (2017). Prevalence of and factors associated with adopting bone health promoting behaviours among people with osteoporosis in Taiwan: a cross-sectional study. BMJ Open.

[11] Turner JG, Gilchrist NL, Ayling EM, Hassall AJ, Hooke EA, Sadler WA (1992). Factors affecting bone mineral density in high school girls. N Z Med J.

[12] Alasagheirin MH, Clark MK (2018). Skeletal growth, body composition, and metabolic risk among North Sudanese immigrant children. Public Health Nurs.

[13] Anagnostis P, Florentin M, Livadas S, Lambrinoudaki I, Goulis DG (2022). Bone health in patients with dyslipidemias: an underestimated aspect. Int J Mol Sci.

[14] McGovern CM, Militello LK, Arcoleo KJ, Melnyk BM (2018). Factors associated with healthy lifestyle behaviors among adolescents. J Pediatr Health Care.

[15] Ng JS, Chin KY (2021). Potential mechanisms linking psychological stress to bone health. Int J Med Sci.

[16] Sagara T, Nishijo M, Hirokawa W, Morikawa Y, Miura K, Tabata M, Nakagawa H (2002). The effects of nutrition and life-style on calcaneal bone mass in high school students [Article in Japanese]. Nihon Koshu Eisei Zasshi.

[17] von Elm E, Altman DG, Egger M, Pocock SJ, Gøtzsche PC, Vandenbroucke JP (2007). The Strengthening the Reporting of Observational Studies in Epidemiology (STROBE) statement: guidelines for reporting observational studies. Ann Intern Med.

[18] Hashimoto K (2005). Reliability and validity of Kasari's modified physical activity index. Kyushu Jpn J Sport Psychol.

[19] Oka K (2003). Stages of change for exercise behavior and self-efficacy for exercise among middle-aged adults [Article in Japanese]. Nihon Koshu Eisei Zasshi.

[20] Tsukahara N, Ezawa I, Miyasita S, Nishimura N, Hayashi Y (1994). Nutritional guidance for osteoporosis prevention: grasping of bone mineral content and nutritional status in a large-scale survey. J Prev Med.

[21] Khan AW, Zadran N, Khan A, Ishaq M, Kumar J, Ibrar A, Tahir A (2020). Vitamin D levels and bone mineral density in premenopausal women compared to postmenopausal women: a multi-centre study from Pakistan. Cureus.

[22] Jiang BC, Villareal DT (2019). Weight loss-induced reduction of bone mineral density in older adults with obesity. J Nutr Gerontol Geriatr.

[23] Miura M, Kawaoka F (2008). Development of a school stressor scale for high school students (SSS). Jpn J Counsell Sci.

[24] Goto R, Ohtsuka R (1999). The relationship between women's bone mineral density and the number of childbirths: an analysis of health examination data in Saitama Prefecture, Japan. Minzoku Eisei.

[25] Yu I, Yamamoto I, Takada OY, Morita R (1992). Bone mass evaluation method using ultrasound: experience using Achilles, a calcaneal ultrasound measurement device. Ther Res.

[26] Troy KL, Mancuso ME, Butler TA, Johnson JE (2018). Exercise early and often: effects of physical activity and exercise on women's bone health. Int J Environ Res Public Health.

[27] Jindo T, Suzukawa K, Kai Y, Kitano N, Osanai H, Ochi E, Nagamatsu T (2018). Association between continuous exercise and sports activity and generalized self-efficacy in male senior high school students: focused on belonging to sports club. Jpn J Hum Growth Dev Res.

[28] Nakazawa A (2014). Seeing sports as educational activities: a postwar history of extracurricular sports activities in Japan. Hitotsubashi J Soc Stud.

[29] Oda H (2016). A case study of local community sports club for children in US. Bulletin of Chiba Keiai Junior College.

[30] Nguyen VH (2018). School-based exercise interventions effectively increase bone mineralization in children and adolescents. Osteoporos Sarcopenia.

[31] Iwaniec UT, Turner RT (2016). Influence of body weight on bone mass, architecture and turnover. J Endocrinol.

[32] Gkastaris K, Goulis DG, Potoupnis M, Anastasilakis AD, Kapetanos G (2020). Obesity, osteoporosis and bone metabolism. J Musculoskelet Neuronal Interact.

[33] Ouyang Y, Quan Y, Guo C (2022). Saturation effect of body mass index on bone mineral density in adolescents of different ages: a population-based study. Front Endocrinol (Lausanne).

[34] Kessler J, Koebnick C, Smith N, Adams A (2013). Childhood obesity is associated with increased risk of most lower extremity fractures. Clin Orthop Relat Res.

[35] López-Peralta S, Romero-Velarde E, Vásquez-Garibay EM, González-Hita M, Robles-Robles LC, Ruiz-González FJ, Pérez-Romero MA (2022). Bone mineral density and body composition in normal weight, overweight and obese children. BMC Pediatr.

[36] Rinonapoli G, Pace V, Ruggiero C, Ceccarini P, Bisaccia M, Meccariello L, Caraffa A (2021). Obesity and bone: a complex relationship. Int J Mol Sci.

[37] Han CS, Kim HK, Kim S (2021). Effects of adolescents' lifestyle habits and body composition on bone mineral density. Int J Environ Res Public Health.

[38] Cadogan J, Eastell R, Jones N, Barker ME (1997). Milk intake and bone mineral acquisition in adolescent girls: randomised, controlled intervention trial. BMJ.

[39] Torres-Costoso A, López-Muñoz P, Ferri-Morales A, Bravo-Morales E, Martínez-Vizcaíno V, Garrido-Miguel M (2019). Body mass index, lean mass, and body fat percentage as mediators of the relationship between milk consumption and bone health in young adults. Nutrients.

[40] Bolland MJ, Leung W, Tai V, Bastin S, Gamble GD, Grey A, Reid IR (2015). Calcium intake and risk of fracture: systematic review. BMJ.

[41] Angbratt M, Timpka T, Blomberg C, Kronhed AC, Waller J, Wingren G, Möller M (2007). Prevalence and correlates of insufficient calcium intake in a Swedish population. Public Health Nurs.

[42] Aslam H, Holloway-Kew KL, Mohebbi M, Jacka FN, Pasco JA (2019). Association between dairy intake and fracture in an Australian-based cohort of women: a prospective study. BMJ Open.

[43] Sim M, Lewis JR, Prince RL (2020). The effects of vitamin K-rich green leafy vegetables on bone metabolism: a 4-week randomised controlled trial in middle-aged and older individuals. Bone Rep.

[44] Ricci JM, Clevenger KA, Sellers S, Davenport S, Pfeiffer KA (2020). Associations between extracurricular activity participation and health-related variables in underrepresented children. Sports Med Health Sci.

[45] Yamasaki S, Kawasaki H, Cui Z (2023). Use of caffeine-containing energy drinks by Japanese middle school students: a cross-sectional study of related factors. Nutrients.

